# Genome‐Wide Identification and Structural Characterization of Secondary Metabolite Biosynthetic Gene Clusters in African Baobab (*Adansonia digitata*)

**DOI:** 10.1002/cbdv.71794

**Published:** 2026-09-30

**Authors:** Ahmed A. Elshikh, Ahmed S. Kabbashi, Imtenan M. Musa

**Affiliations:** ^1^ Laboratory of Genetic Diversity and Evolution (LAGEVol) Department of Biology Center for Human and Biological Sciences Federal University of São Carlos (UFSCar) São Paulo Brazil; ^2^ Department of Botany, Faculty of Science and Technology Omdurman Islamic University Omdurman, Khartoum Sudan; ^3^ Department of Biomedical Science Faculty of Pharmacy Omar Al‐Mukhtar University Al Bayda' Libya; ^4^ Department of Microbiology and Parasitology Medicinal and Aromatic Plants and Traditional Medicine Research Institute National Center for Research Khartoum Sudan; ^5^ Department of Parasitology Faculty of Medical Laboratory Sciences International University of Africa Khartoum Sudan; ^6^ Department of Biotechnology Faculty of Science and Technology Omdurman Islamic University Omdurman, Khartoum Sudan

**Keywords:** *Adansonia digitata*, biosynthetic gene clusters (BGCs), BURP domain, genome mining, plantiSMASH, secondary metabolites

## Abstract

*Adansonia digitata* L., known as the African Baobab, is renowned for its extraordinary longevity and its long‐standing use in traditional African medicine. However, the genomic basis underlying its specialized secondary metabolism remains largely unexplored. This study aimed to computationally identify and characterize putative biosynthetic gene clusters (BGCs) within the publicly available *A. digitata* genome assembly (GenBank accession GCA_029448705.1) using an integrated bioinformatics pipeline. Ab initio gene structure prediction was performed with AUGUSTUS (*Arabidopsis thaliana* species model), functional protein domains were annotated with InterProScan, and candidate BGCs were mined using the plantiSMASH 2.0 webserver. Gene prediction yielded 52 721 protein‐coding gene models genome‐wide, of which 45 641 (86.6%) received at least one InterProScan domain annotation. The analysis identified 50 putative BGCs distributed across 13 scaffolds, ranging from 15.3 to 492.8 kb in size and provisionally classified into eight chemical categories, most frequently saccharide‐type (*n* = 20) and cyclopeptide‐type (*n* = 9) clusters. Notably, nine clusters carried a BURP‐domain core gene consistent with a cyclopeptide/defense‐peptide‐associated pathway, including the single largest cluster identified (492.8 kb); other clusters carried core domains consistent with terpene synthase, squalene‐hopene cyclase, chalcone/stilbene synthase (Chal_sti_synt_N), and dirigent‐protein/lignan‐associated biosynthesis. None of the 50 clusters showed a significant match to a characterized reference cluster in the MIBiG database, suggesting these represent largely uncharacterized biosynthetic loci. These results represent in silico predictions rather than experimentally confirmed metabolic functions, since no transcriptomic, metabolomic, or biochemical validation was performed as part of this study. Nonetheless, this work provides one of the first genome‐wide catalogues of putative BGCs in *A. digitata* and offers a preliminary resource to guide future comparative genomics, expression profiling, and experimental validation of specialized metabolism in this species.

## Graphical abstract

Genome‐wide computational analysis of the African baobab (*Adansonia digitata*) identifies 50 candidate biosynthetic gene clusters spanning terpene, polyketide, cyclopeptide, and lignan pathways, including nine BURP‐domain clusters linked to putative chemical defense. Integrating AUGUSTUS gene prediction, InterProScan domain annotation, and plantiSMASH mining provides the first genome‐wide catalogue of *A. digitata*'s specialized‐metabolite gene clusters, offering a resource for future comparative genomics and experimental validation.

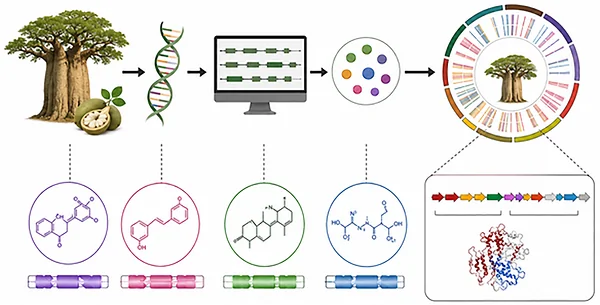

## 1 Introduction

*Adansonia digitata* L., a prominent member of the Malvaceae family, is a massive, long‐lived deciduous tree native to the arid regions of Central Africa. Widely distributed across sub‐Saharan Africa, it thrives in semi‐arid and semi‐humid climates [1]. Morphologically, the species is characterized by a swollen, thick trunk that can reach up to 10 m in diameter, often exhibiting a distinct bottle‐like shape. Its smooth bark ranges from reddish‐brown to gray, protecting an extensive lateral root system that terminates in tubers. The tree bears large, solitary, drooping flowers and indehiscent woody fruits containing numerous seeds embedded in a naturally dry, acidic pulp [2]. The baobab serves as a vital nutraceutical and therapeutic resource in Africa. Its fruit pulp is utilized in formulating beverages, confectionery, and the traditional “baobab milk,” and is characterized by its long shelf life when stored as a powder. The protein‐rich leaves serve as a dietary staple, consumed either fresh as a vegetable or dried for use in soups. At the same time, the seeds are processed as a coffee substitute or incorporated into traditional cuisines. From a pharmacological perspective, the pulp is employed to treat febrile illnesses, dysentery, and measles, while root‐derived preparations serve as a tonic for malaria patients, and the bark‐derived gum is applied to accelerate wound healing. Beyond its utility as high‐value livestock fodder, the tree's hydrological significance is underscored by its capacity to sequester substantial volumes of rainwater within its hollow trunks, acting as a critical lifeline in arid ecosystems [3]. *A. digitata* is a rich reservoir of bioactive metabolites, notably polyphenols such as gallic acid, quercetin, and various procyanidins (A2, B2, B5, and C1). These compounds are renowned for their antioxidant and insulin‐sensitizing properties, contributing to the management of metabolic disorders. The pulp also contains high concentrations of vitamin C, triterpenes, and saponins, alongside dietary fibers like pectin. Despite the presence of certain antinutrients (e.g., tannins and phytic acid), the fruit's nutritional value remains significant. However, this phytochemical composition is highly variable, influenced by the complex interplay of genetic factors, environmental conditions, and postharvest handling [4]. Recent genomic advancements have significantly expanded our understanding of *A. digitata*. High‐resolution studies, including the assembly of its chromosome‐level genome, have provided profound insights into its evolutionary history and complex polyploid nature [5]. Molecular characterization and DNA barcoding utilizing multi‐loci regions such as ITS, rbcL, and psbA‐trnH have further refined the species' identification and phylogeography, suggesting a West African center of origin [6, 7]. Moreover, cyto‐molecular analyses have recently updated the chromosome number and structural features of this *A. digitata* [8]. Despite these significant advancements in structural genomics and phylogenetic mapping, the functional genomic landscape of *A. digitata* specialized metabolism remains a critical black box. While the tree is known to synthesize a sophisticated array of bioactive compounds such as antioxidant polyphenols and antimicrobial triterpenes, the biosynthetic logic and genomic clustering of the genes responsible for these metabolites have not been elucidated. In plants, genes involved in specialized pathways are often organized into biosynthetic gene clusters (BGCs), a feature that facilitates coordinated inheritance and regulation. Characterizing these clusters is essential not only for understanding the tree's exceptional chemical defense and extreme longevity but also for unlocking its potential in metabolic engineering and drug discovery. Genome mining for plant BGCs has advanced considerably in recent years. Beyond gene‐cluster identification, recent work has increasingly focused on the ecological, regulatory, and evolutionary basis of BGC formation and maintenance in plant genomes, including their association with distinct chromatin signatures and transposable‐element‐rich genomic regions that facilitate coordinated, stress‐ and tissue‐specific expression [9]. In parallel, the computational tools underlying plant genome mining continue to be actively developed: antiSMASH, the platform on which plantiSMASH is built, has been substantially updated to improve detection, regulatory element prediction, and chemical structure visualization [10], and plantiSMASH itself has recently been updated to plantiSMASH 2.0, with improved detection, annotation, and prioritization of candidate plant BGCs [11]. Therefore, this study provides the first genome‐wide identification and functional characterization of BGCs in *A. digitata*, leveraging high‐resolution gene prediction and automated metabolic mining to bridge the gap between its complex genome and its renowned pharmacological profile.

## 2 Materials and Methods

### 2.1 Genome Data Acquisition

The reference genome assembly of *A. digitata* was retrieved from the National Center for Biotechnology Information (NCBI) database. The specific assembly used was ASM2944870v1 (GenBank accession: GCA_029448705.1), derived from isolate IRGN CZY4BLHFFT and submitted by Iridian Genomes in March 2023. The genome comprises scaffold‐level sequences representing the eudicot genome of the Malvaceae family (taxonomy ID: 69109). This assembly (GCA_029448705.1, scaffold prefix JAQSVH01, biosample ID SAMN3062256) corresponds to the genome sequence originally generated and described by [12]. The assembly characteristics reveal a total genome size of 687 Mb (with a total ungapped length of 673.5 Mb and a GC content of 31.5%), distributed across 232 407 scaffolds (scaffold N50 of 8.9 kb and L50 of 7756) and 280 892 contigs (contig N50 of 4.6 kb and L50 of 35 030), achieving a genome coverage depth of 110x at the scaffold assembly level. The present study reanalyzes these publicly available data via NCBI GenBank and does not report any new sequencing; we gratefully acknowledge the original generators of this genomic resource.

### 2.2 Genome Annotation and Protein Prediction

Coding sequences within the *A. digitata* genome assembly were predicted using AUGUSTUS (version 3.5.0), a eukaryotic ab initio gene‐prediction tool that uses a generalized hidden Markov model to identify open reading frames (ORFs) from genomic DNA sequence alone. The AUGUSTUS run using the built‐in *Arabidopsis thaliana* (arabidopsis) species/parameter set. Confirmed job parameters were: built‐in training set; softmasking enabled; gene prediction on both strands; complete gene model; extrinsic hints/evidence disabled (–hints = false); UTR prediction disabled; in‐frame stop codons not permitted; and output limited to predicted protein sequences, coding sequence, and CDS coordinates. This is acknowledged as a methodological limitation of the present study (see Section 5): even with an appropriate species model, pure ab initio prediction without RNA‐seq or protein‐homology evidence can generate false‐positive gene models and imprecise exon‐intron boundaries, particularly in structurally complex or recently duplicated genomic regions, which are common in polyploid genomes such as that of *A. digitata* [5]. The ab initio‐only approach was adopted because no matched RNA‐seq dataset was available for this *A. digitata* genome assembly at the time of analysis, precluding evidence‐guided annotation. Future work should incorporate transcriptomic evidence (e.g., via BRAKER2) and/or protein‐homology evidence (e.g., via MAKER or EvidenceModeler) to further improve the accuracy and completeness of gene models, particularly for candidate biosynthetic loci.

### 2.3 Functional Annotation and Domain Identification

Functional annotation of predicted protein sequences (52 721 sequences), derived from the *A. thaliana* model using AUGUSTUS, was performed using InterProScan (version 5.59–91.0). The analysis incorporated eighteen databases and tools, including TIGRFAM, FunFam, SFLD, SUPERFAMILY, PANTHER, Gene3D, HAMAP, PROSITE Profiles, coils, SMART, CDD, PRINTS, PIRSR, PROSITE patterns, AntiFam, Pfam, MobiDBLite, and PIRSF. Options for Gene Ontology (GO) term assignment and pathway annotation were enabled, without the use of any proprietary applications.

Outputs were generated in three parallel formats to ensure compatibility with subsequent analyses: Tab‐separated values (TSV; approximately 587 548 rows and 15 columns), GFF3 (∼1.0 million rows and 9 columns; 2.8 GB), and XML. These outputs were utilized to support both tabular domain summaries and the direct import of gene/domain coordinates for downstream visualization (Section 2.5). Among the 52 721 protein models, 45 641 (86.6%) were annotated with at least one domain. This count was determined by identifying unique protein/gene identifiers (Column 1) in the TSV file using the “Unique” tool available on the Galaxy platform.

### 2.4 Identification of Specialized Metabolite Biosynthetic Gene Clusters (BGCs)

To mine specialized metabolic pathways, the annotated proteome was screened using the plantiSMASH 2.0 web server (plantismash.bioinformatics.nl). Candidate BGCs were identified by detecting core biosynthetic enzyme domains (e.g., terpene synthases and polyketide synthases) flanked by tailoring enzymes (e.g., cytochrome P450s and glycosyltransferases) and categorized into provisional chemical classes (Table S1). All predictions are based purely on computational genome mining, with inferred products reflecting the domain architecture of the core gene rather than confirmed metabolites.

To map plantiSMASH‐reported core domains to specific genomic loci, AUGUSTUS‐derived gene coordinates were intersected with each cluster's interval (Table S1). Core genes were unambiguously assigned for clusters featuring rare, distinctive domains (e.g., BURP, Dirigent, Terpene synthase, squalene‐hopene cyclase, and Chal_sti_synt_N) via matching InterProScan annotations (Section 2.3). For clusters driven by common, multi‐copy domains (e.g., p450, Epimerase, and UDPGT_2), all matching candidate genes across the 50 clusters were retained.

Based on mapping ambiguity, each BGC was assigned a confidence tier: high (unique rare‐domain match), low‐to‐moderate (multiple candidate matches for common domains), or not resolved (no matching annotation). These tiers and identified core genes are detailed in Table S1. Five high‐confidence core genes representing major structural classes were selected for structural visualization using GSDS 2.0 as Figure 3 (Section 2.5).

### 2.5 Gene Structure Visualization

Exon‐intron structural diagrams for representative core genes (Figures 1 and 3) were generated using the Gene Structure Display Server 2.0 (GSDS 2.0; [13]). To ensure consistent architectural rendering, gene coordinates were extracted from the AUGUSTUS GTF/GFF3 output and compiled into a single unified file. This consolidated dataset was submitted to GSDS 2.0, allowing for the batch visualization of all selected genes in a single operation.

**FIGURE 1:**
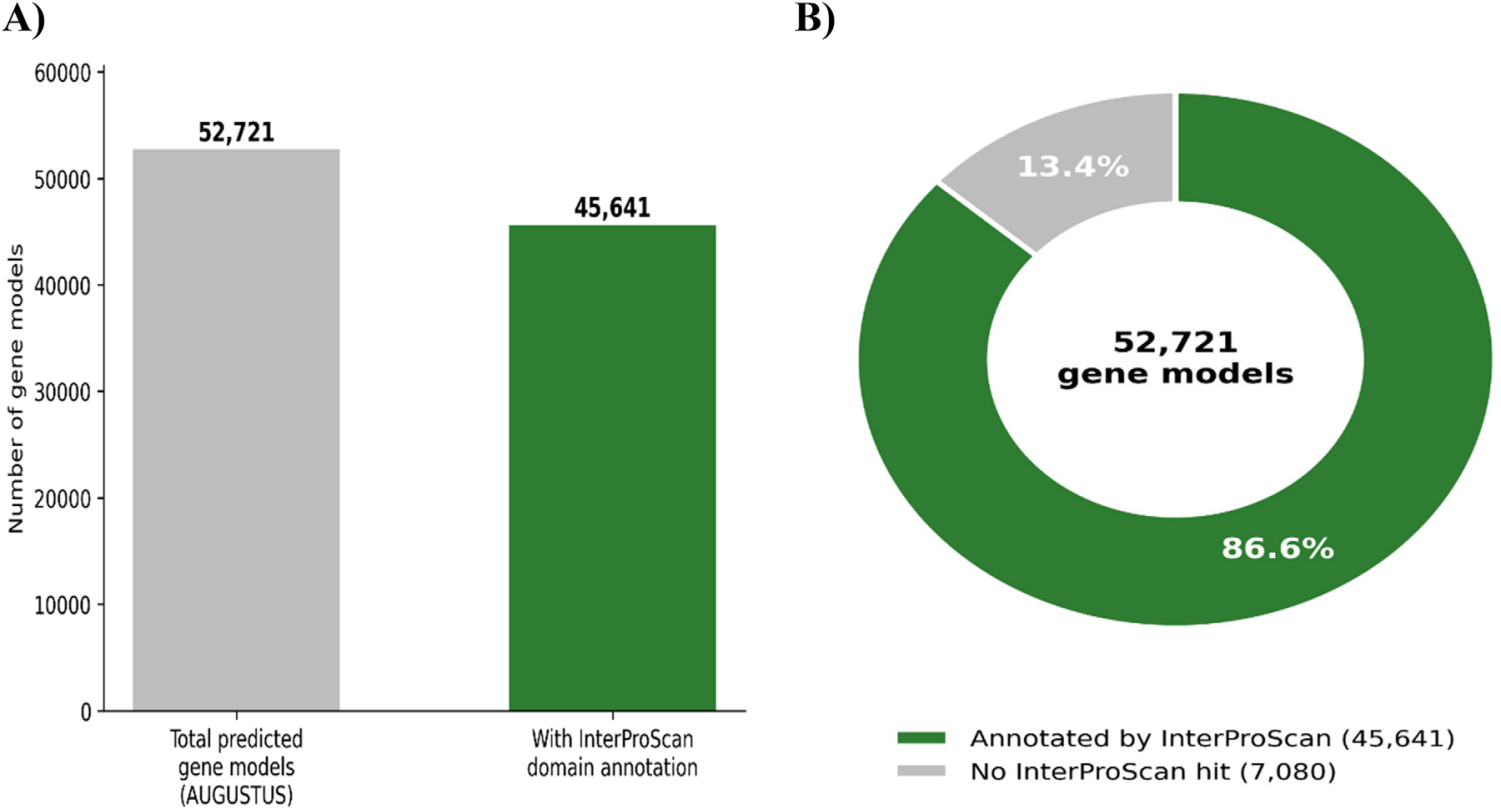
Completeness of functional annotation of the AUGUSTUS‐predicted gene set. (A) Total number of protein‐coding gene models predicted by AUGUSTUS genome‐wide (52 721) compared with the number receiving at least one InterProScan v5.59–91.0 domain annotation (45 641). (B) Proportion of predicted gene models with (86.6%) versus without (13.4%, *n* = 7080) an InterProScan domain hit.

### 2.6 Hardware and Software Environment

All computational analyses in this study were performed using public web‐based bioinformatics platforms rather than local computing infrastructure: ab initio gene prediction was run as an AUGUSTUS tool job on the Galaxy Europe server (usegalaxy.eu); functional annotation used InterProScan Galaxy tool version 5.59–91.0; BGC mining was performed on the plantiSMASH 2.0 public webserver (plantismash.bioinformatics.nl); and gene‐structure figures were generated using GSDS 2.0 (gsds.gao‐lab.org).

### 2.7 Statistical Analysis and Data Visualization

This study is a descriptive, single‐genome computational annotation and genome‐mining analysis that does not involve biological replicates, treatment groups, or physical experimental measurements; consequently, inferential statistical testing (e.g., *t*‐tests or ANOVA) did not apply to the primary workflow. Quantitative summary metrics, including total gene counts, functional domain distributions, and cluster size statistics, were compiled, structured, and curated using spreadsheet software (Microsoft Excel). Furthermore, data aggregation, descriptive statistical summaries, and graphical visualizations were executed and rendered using the R statistical computing environment. All reported findings represent descriptive metrics derived directly from the computational and curation pipelines.

## 3 Results

Gene prediction with the (*A. thaliana* model) AUGUSTUS run identified a total of 52 721 predicted protein‐coding gene models genome‐wide. Of these, 45 641 (86.6%) received at least one InterProScan v5.59–91.0 domain annotation (Section 2.3; Figure 1).

Candidate BGCs (Figure 2, Table S1) were distributed across 13 of the genome assembly scaffolds, indicating that biosynthetic‐gene clustering is not confined to a single genomic region. Genome mining using the plantiSMASH 2.0 web server, run on the *A. thaliana* model gene/protein set, identified 50 candidate BGCs distributed across 13 scaffolds (Figure 2). Cluster sizes ranged from 15.3 to 492.8 kb. Clusters were classified by plantiSMASH into eight provisional chemical categories: saccharide (*n* = 20), cyclopeptide (*n* = 9), alkaloid (*n* = 6), terpene (*n* = 6), putative (*n* = 5), polyketide (*n* = 2), lignan (*n* = 1), and a mixed saccharide‐alkaloid cluster (*n* = 1) (Figure 2). None of the 50 clusters showed a statistically significant match to a characterized reference cluster in the MIBiG database under the default plantiSMASH similarity search, indicating that these candidate clusters are not closely related to currently curated, experimentally characterized plant BGCs.

**FIGURE 2:**
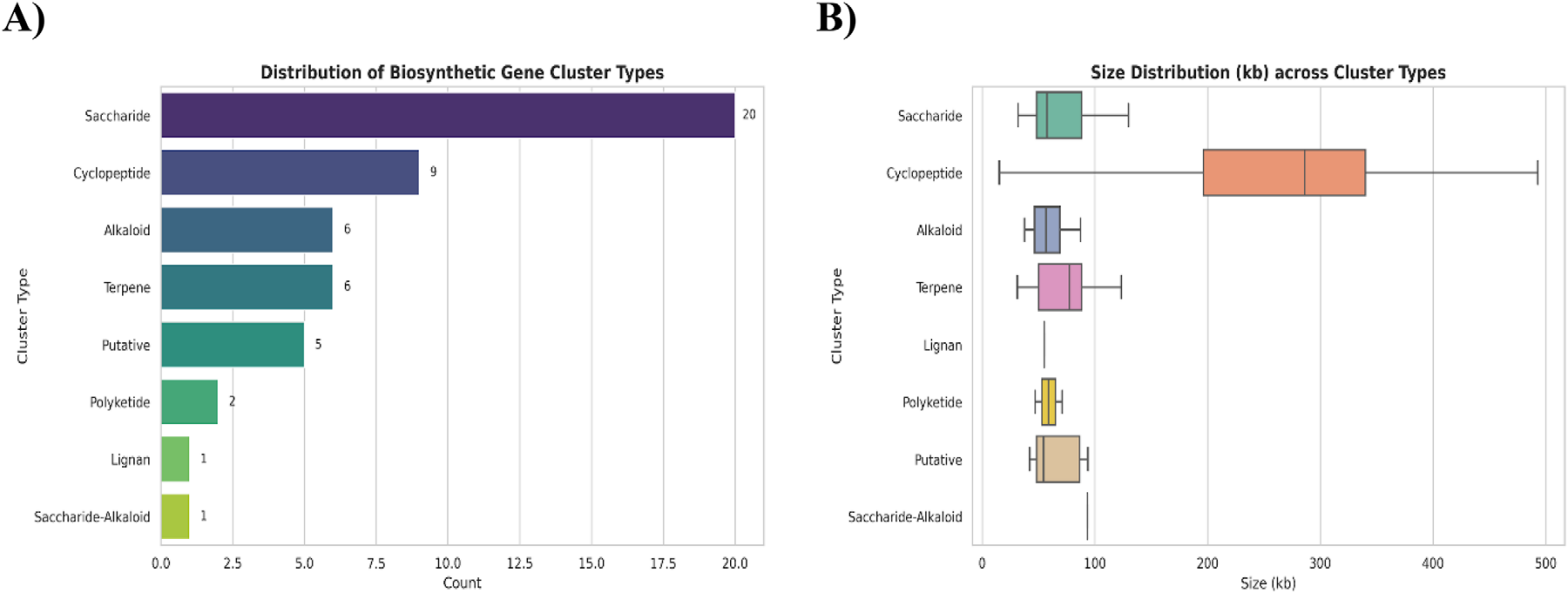
Summary of the 50 candidate BGCs identified by plantiSMASH 2.0. (A) Number of candidate BGCs per provisional chemical class. (B) Size distribution (kb) of candidate BGCs by chemical class, shown as box‐and‐whisker plots (box: interquartile range; center line: median; whiskers: full range). The full cluster‐by‐cluster listing (scaffold, coordinates, size, core domains, predicted subgroup, and MIBiG similarity for all 50 clusters) is provided in Table S1.

The nine cyclopeptide‐type clusters were each defined by a BURP‐domain core gene and were found on six different scaffolds; this class included the single largest cluster identified in the genome (492.8 kb) and showed the widest size range of any class (Figure 2B). The six terpene‐type clusters included two containing terpene synthase domains (terpene synth/terpene synth C) and two containing squalene‐hopene cyclase domains (SQHop_cyclase_N/C), consistent with monoterpene/sesquiterpene‐ and triterpenoid/saponin‐associated pathways, respectively. The two polyketide‐type clusters both contained a Chal_sti_synt_N domain, the shared N‐terminal domain of the chalcone synthase/stilbene synthase enzyme family, consistent with a role in flavonoid or stilbenoid biosynthesis, though this domain alone does not distinguish between the two activities. The single lignan‐type cluster contained a dirigent‐domain gene together with Aldo_ket_red and SQS_PSY domains, consistent with a lignan‐biosynthesis‐associated gene arrangement. The structural organization and exon‐intron architecture of representative core genes from selected clusters are shown in Figure 3.

**FIGURE 3:**
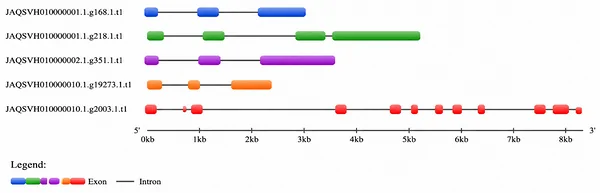
Structural organization of five representative core genes spanning the major BGC classes identified in *A. digitata*. Exon‐intron structure was generated using GSDS 2.0 [13] from the AUGUSTUS gene models; all five genes were submitted in a single batch to ensure a consistent, gene‐specific color scheme. From top to bottom: JAQSVH010000001.1.g1683 (Cluster 6, Terpene‐type; core Terpene synthase domain); JAQSVH010000001.1.g2188 (Cluster 8, lignan‐type; core dirigent domain); JAQSVH010000002.1.g3517 (Cluster 10, polyketide‐type; core Chal_sti_synt_N domain, chalcone/stilbene synthase family); JAQSVH010000010.1.g19213 (Cluster 30, cyclopeptide‐type; core BURP domain); and JAQSVH010000010.1.g20043 (Cluster 33, terpene‐type; core squalene‐hopene cyclase domain). Full gene coordinates and cluster assignments are given in Table S1. Exons are shown as filled blocks (color denotes gene identity, not domain type) and introns as connecting lines; scale bar in kilobases (kb).

Cross‐referencing InterProScan domain annotations with plantiSMASH cluster coordinates (Section 2.4) resolved at least one candidate core gene for 41 of the 50 clusters (Table S1). Sixteen clusters—all nine BURP‐domain cyclopeptide‐type clusters, the dirigent‐domain lignan‐type cluster, two terpene synthase‐domain clusters, two squalene‐hopene cyclase‐domain clusters, and both Chal_sti_synt_N‐domain polyketide‐type clusters—were resolved with high confidence, each core domain matching one or a small number of genes uniquely within its cluster. A further 25 clusters, defined by common domains shared across large gene families (e.g., p450, Epimerase, “SE”), were resolved only to a list of candidate genes (up to 14 candidates for a single cluster), reflecting low‐to‐moderate confidence rather than a single confirmed core gene. The remaining nine clusters returned no matching gene and are unresolved. Notably, Clusters 21 and 22 (both BURP‐domain, on scaffold JAQSVH010000008.1) were found to share the same four core genes and have substantially overlapping genomic coordinates (351 117–547 407 bp and 374 317–615 392 bp, respectively), suggesting they may represent a single underlying locus rather than two independent clusters.

## 4 Discussion

The computational identification of 50 candidate BGCs in the *A. digitata* genome is consistent with the hypothesis of substantial genomic investment in specialized metabolism in this species. This number is broadly consistent with the recent chromosome‐level assembly reported by [5], who described two whole‐genome duplication (WGD) events in the baobab lineage. WGD is known to facilitate the expansion of gene families and could plausibly provide raw material for the diversification of biosynthetic pathways, although a direct causal link between the WGD events and the specific clusters identified here was not tested in the present study and remains a hypothesis for future investigation.

Among the identified clusters, the nine BURP‐domain cyclopeptide‐type clusters are of particular interest: BURP‐domain proteins have been reported to participate in plant stress and defense responses in other species, and the recurrence of this domain across nine independent cluster calls on six scaffolds—including the single largest cluster identified (492.8 kb)—raises the hypothesis that this gene family has undergone substantial expansion in *A. digitata*, tentatively consistent with previously reported antimicrobial and defense‐related activity of baobab extracts [14]. Gene‐level cross‐referencing of InterProScan domain calls against plantiSMASH cluster boundaries (Section 2.4) identified the specific core BURP‐domain gene for each of these clusters (e.g., g515 for Cluster 2, g1138 for Cluster 4, g19213 for Cluster 30; full list in Table S1), and additionally revealed that Clusters 21 and 22 share the same four core genes and substantially overlapping coordinates, indicating these likely represent a single genomic locus detected twice rather than two independent BGCs; we therefore consider the biologically meaningful count of distinct BURP‐associated loci to be eight, not nine. The two Chal_sti_synt_N‐domain Polyketide‐type clusters offer a plausible, though still hypothetical, molecular basis that is broadly consistent with previously reported polyphenol content in baobab fruit pulp [15] and with the broader pharmacological interest in Malvaceae secondary metabolites [16]; however, because the Chal_sti_synt_N domain is shared between chalcone synthase and stilbene synthase, this analysis alone cannot distinguish which specific activity (or both) these loci encode. We emphasize that all of these associations are inferred from domain architecture alone; confirming that these genes are transcribed, translated, and enzymatically active in planta will require future transcriptomic and/or biochemical validation.

The absence of any significant MIBiG match across all 50 candidate clusters is itself noteworthy: it suggests that *A. digitata* specialized‐metabolite gene clusters, as currently predicted, are not closely related to the reference biosynthetic pathways curated from other, better‐studied plant species. This could reflect genuinely novel or lineage‐specific chemistry, consistent with the taxonomically isolated position of Adansonia within the Malvaceae, but could equally reflect current gaps in MIBiG's coverage of non‐model plant biosynthesis, or residual inaccuracies in the underlying gene models. We are unable to distinguish between these explanations with the present data.

The presence of terpene synthase‐ and squalene‐hopene‐cyclase‐domain‐containing terpene‐type clusters, together with a dirigent‐domain lignan‐type cluster, is broadly consistent with the general chemical diversity previously reported across the Malvaceae family [17]. We caution, however, that the present analysis was restricted to a single *A. digitata* genome assembly. Given the reported polyploid, structurally complex nature of the baobab genome [5] and known intraspecific genetic diversity in *A. digitata* [6, 7], the BGC repertoire described here should not be generalized as representative of the species as a whole; additional assemblies from independent individuals/populations would be required to assess how conserved these clusters are within the species.

A direct, cluster‐by‐cluster comparison with related Malvaceae genomes (e.g., *Theobroma cacao*, *Gossypium* spp., and *Hibiscus* spp.) was outside the scope of the present analysis and was not performed. Reference genomic resources are available for at least one close relative (the *T. cacao* genome; [18]), and we identify formal comparative BGC mining across these Malvaceae genomes as a priority direction for future work, which would help clarify whether the clusters reported here are broadly conserved across the family or represent baobab‐specific expansions.

### 4.1 Limitations

Status: the ab initio gene prediction was run with AUGUSTUS species model (*A. thaliana*; see Section 2.2); plantiSMASH BGC mining is complete on this gene set (Figure 2, Table S1, 50 clusters). Candidate core genes were identified for 41 of the 50 clusters by cross‐referencing InterProScan domain calls with cluster coordinates (Section 2.4): 16 with high confidence (rare, distinctive core domains matching a unique gene) and 25 with only low‐to‐moderate confidence (common domains matching multiple candidate genes within the cluster, so the true core gene cannot be distinguished computationally); 9 clusters remain fully unresolved. Figure 3 has been generated using five of the high‐confidence core genes. Figures 1 and 2 are finalized; only minor methods details (BUSCO, exact plantiSMASH cluster‐definition parameters) remain outstanding.

Beyond this, gene prediction relied on ab initio modeling without RNA‐seq or protein‐homology evidence (no matched RNA‐seq dataset was available for this *A. digitata* assembly at the time of analysis), which may further affect the accuracy of individual gene models. BGC identification and classification are based entirely on computational domain co‐occurrence and have not been validated by transcriptomic, metabolomic, or biochemical experiments; predicted “products” are therefore best understood as hypotheses rather than confirmed metabolites. Relatedly, the genomic presence of a candidate BGC indicates biosynthetic potential only and does not by itself demonstrate that the corresponding genes are actively transcribed or that the predicted metabolite is produced under natural conditions: plant BGC expression is frequently tissue‐specific, developmental‐stage‐specific, or induced only under particular environmental or stress conditions [9], so confirming active expression and metabolite production will require tissue‐specific transcriptomic and/or metabolomic profiling under relevant physiological conditions, rather than genome sequence data alone. Core‐gene assignment for clusters defined by common, multi‐copy protein domains (e.g., p450, Epimerase) is inherently less certain than for clusters defined by rare, distinctive domains, since several candidate genes within the same cluster window often carry a matching annotation; Table S1 reports a confidence tier for every cluster so this limitation is transparent on a cluster‐by‐cluster basis, and nine clusters have no computationally resolved core gene at all. As noted above, Clusters 21 and 22 were found to share identical core genes and overlapping coordinates, illustrating that plantiSMASH cluster‐calling can occasionally split a single locus into adjacent, overlapping cluster entries; we did not systematically screen all 50 clusters for this artifact, so a small number of additional redundant calls cannot be excluded. The analysis is based on a single reference genome assembly and does not capture intraspecific variation in BGC content, and no formal comparative genomic analysis with related Malvaceae species was performed. Addressing these limitations through RNA‐seq‐informed annotation, multi‐genome comparison, and targeted experimental validation (e.g., qRT‐PCR expression profiling or metabolite profiling of tissues predicted to express candidate clusters) represents a clear direction for future work.

## 5 Conclusion

This study presents a computational genome‐mining analysis of secondary metabolism in *A. digitata*. Using an integrated pipeline combining AUGUSTUS for gene structure prediction and plantiSMASH 2.0 for pathway mining, we identified 50 candidate BGCs distributed across 13 scaffolds of the available genome assembly, ranging from 15.3 to 492.8 kb in size. The recurrence of BURP‐domain core genes across nine independent clusters, including the largest cluster identified, is consistent with the hypothesis that this gene family has undergone notable expansion in *A. digitata* and may contribute to chemical defense, while two Chal_sti_synt_N‐domain clusters raise a testable hypothesis regarding flavonoid/stilbenoid biosynthesis, and additional terpene synthase‐, squalene‐hopene‐cyclase‐, and dirigent‐domain‐containing clusters point to further diversity in terpenoid and lignan‐associated pathways. None of the 50 clusters matched a characterized reference cluster in the MIBiG database, suggesting these represent largely uncharacterized biosynthetic loci. These findings should be regarded as a preliminary, hypothesis‐generating catalogue rather than definitive evidence of biosynthetic function, given the absence of transcriptomic, metabolomic, or experimental validation and the single‐genome scope of the analysis. We suggest that this catalogue provides a useful starting resource for future comparative genomics across the Malvaceae, expression profiling, and targeted experimental validation, which together could help clarify the genetic basis of *A. digitata*’s chemical diversity and its potential relevance to metabolic engineering and natural‐product discovery.

## Author Contributions

**Ahmed A. Elshikh**: conceptualization, methodology, software, formal analysis, writing – original draft, and writing – review and editing. **Ahmed S. Kabbashi**: methodology, validation, and writing – review and editing. **Imtenan M. Musa**: writing – original draft, software, and writing – review and editing .

## Funding

This research received no specific grant from any funding source.

## Conflicts of Interest

The authors declare no conflicts of interest.

## Use of Generative AI and AI‐Assisted Technologies in the Writing Process

During the preparation of this work, the authors used Gemini for language proofreading and assisting in the design of the graphical abstract. After using this tool, the authors reviewed, edited, and verified the content and visuals as needed, and take full responsibility for the content of the publication

## Supporting information




**Supporting File 1**: cbdv71794‐sup‐0001‐SuppMat.xlsx.

## Data Availability

The genome assembly analyzed in this study is publicly available in the NCBI GenBank database under accession GCA_029448705.1 (BioProject associated with [12]). All computational outputs generated in this study (predicted gene models, InterProScan annotations, and plantiSMASH cluster predictions) are provided in Supplementary Table S1 and are available from the corresponding author upon reasonable request.
